# “If I don’t smoke shisha, I won’t be able to sleep”: lived experiences of high school students in Ethiopia

**DOI:** 10.29392/001c.33806

**Published:** 2022-04-25

**Authors:** Selamawit Hirpa, Fiona Dobbie, Andrew Fogarty, Adamu Addissie, Mirgissa Kaba, Thomas Frese, Susanne Unverzagt, Eva Johanna Kantelhardt, Kamran Siddiqi, Linda Bauld, Wakgari Deressa

**Affiliations:** 1 Department of Preventive Medicine, School of Public Health, Addis Ababa University, Addis Ababa, Ethiopia; Institute of General Practice and Family Medicine, Centre of Health Sciences, Martin-Luther-University Halle-Wittenberg, Germany; 2 Usher Institute and SPECTRUM Consortium, University of Edinburgh, UK; 3 Division of Epidemiology and Public Health, University of Nottingham, Nottingham, UK; 4 Department of Preventive Medicine, School of Public Health, Addis Ababa University, Addis Ababa, Ethiopia; Institute for Medical Epidemiology, Biostatistics and Informatics, Centre of Health Sciences, Martin-Luther-University Halle-Wittenberg, Germany; 5 Department of Preventive Medicine, School of Public Health, Addis Ababa University, Addis Ababa, Ethiopia; 6 Institute of General Practice and Family Medicine, Centre of Health Sciences, Martin-Luther-University Halle-Wittenberg, Germany; 7 Institute for Medical Epidemiology, Biostatistics and Informatics, Centre of Health Sciences, Martin-Luther-University Halle-Wittenberg, Germany; 8 The University of York and Hull York Medical School, Health Sciences, UK

**Keywords:** Lived experiences, shisha, high school, students, Ethiopia

## Abstract

**Background:**

Shisha smoking predisposes the users to cardiovascular diseases, cancer, and infections, such as tuberculosis, hepatitis, and herpes. In Ethiopia, there is little data on the adolescents’ shisha smoking experience. This study aimed to explore the lived experience of high school students and inform ongoing and future prevention and control interventions.

**Methods:**

This study was conducted in Addis Ababa and Adama cities in Ethiopia. Twenty-five secondary school students aged 15-22 years who had shisha smoking experience participated in this study. A topic guide was used to facilitate the in-depth interviews (IDIs) and a digital audio recorder recorded the interviews. Interviews varied between 40-90 minutes and were conducted in private open-air spaces where only the interviewee and researcher were present. Each transcript was coded using Atlas.ti version 8 software. The analytical approach was iterative, with interview transcripts analyzed at the time of coding and re-analyzed after a preliminary result was drafted to search for additional themes.

**Results:**

Students described two key factors that influenced their decision to initiate shisha smoking: peer influence and perceiving it as a means to release stress. After initiating shisha use students maintained the behaviour because of: peer influence, khat chewing, enjoyment of shisha smoking, having prolonged leisure time, and accessibility to shisha. Students regretted the impact shisha use had on their lives, such as conflict with their families, poor academic performance, and spending money on shisha smoking. Female students were also concerned about reproductive health risks related to shisha use.

**Conclusions:**

Peer influence played a major role both in initiating and maintaining shisha use. However, students admitted concern over the impact of shisha smoking on academic performance and their relationship with their families. Since shisha use is associated with khat chewing; shisha smoking control programs cannot be successful without controlling khat. Especially young girls had worries about their reproductive health risks associated with shisha use. This suggests that targeted awareness raising programs highlighting the dangers of shisha use for both health and safety; especially for young women is required.

A water pipe that is used to smoke tobacco is known by different names in different countries including hookah, narghile, argileh, shisha, and goza.^1–3^ Shisha is a device for smoking flavoured or non -flavoured tobacco invented in the 16th century by a physician named Hakim Abul-Fatha Gilani.^4^ The purpose of the device is to pass the smoke through water in an attempt to ‘purify’ the smoke before inhaling^1^. Shisha use in Ethiopia is a fairly recent development thought to have been first introduced by Middle Eastern restaurants in 2003.^5^ However, due to popular urban trends and lifestyle changes, most restaurants in the main cities of Ethiopia started serving shisha in the past few years.^5,6^

While the belief that shisha smoking is less harmful than tobacco smoking is prevalent,^4^ studies have found that shisha smoking contributes to cardiovascular diseases, cancer, and infections, such as tuberculosis, hepatitis, and herpes as a result of sharing the mouthpiece.^4,7,8^ Similar to other tobacco products, shisha smoking delivers the addictive drug nicotine, which can lead to dependence and users seeking regular access to nicotine products. Studies have shown that shisha smoking is perceived by users to be less harmful than cigarettes because the smoke passes through water and is filtered.^9,10^ However, the shisha smoker may inhale as much smoke in one session as a cigarette smoker would inhale by smoking 100 or more cigarettes.^7^ Available evidence reveals that reasons given for shisha smoking include, social acceptance, availability, affordability, curiosity, fashion, peer influence, attractive flavours, social stress, and poor academic performance.^9,11,12^ In Ethiopia, in addition to the above-mentioned factors chewing khat has been noted as an entry point for shisha smoking and use of other substances.^13–15^

Based on the Food and Medicine Drug Administration Proclamation 11/12 of 2019, selling, and smoking shisha is forbidden in Ethiopia.^15^ Despite this, it is common to see shisha houses nearby school compounds and shisha services in the hotels or restaurants in Addis Ababa and many cities and towns across the country.^5^,^6^ A review of the literature found few published studies on adolescent shisha smoking in Ethiopia. A qualitative study conducted in 2013 among shisha users in Bahir Dar city reported that accessibility, peer pressure, shisha flavour, lack of knowledge and absence of effective policy were the main reasons for shisha smoking.^12^ In March 2020, we conducted a quantitative study among high school adolescents in Addis Ababa and four regions in Ethiopia and the findings showed that, having friends who smoke shisha and ever use of substances (khat, cigarette, smokeless tobacco, and marijuana) were associated with ever use of shisha.^16^ The purpose of the qualitative study reported in this paper was to explore the lived experiences of shisha smoking among high school students in two cities in Ethiopia. This study will contribute to our understanding of the experiences of shisha smokers and the results may help to inform policymakers to develop strategies to prevent shisha smoking among school children.

## Methods

### Participants and Study Approach

The study was conducted in Addis Ababa (the largest and capital city) and Adama (one of the major cities) in Ethiopia. The inclusion criteria for this qualitative study were students who attended selected high schools which were part of the afore-mentioned quantitative school survey for shisha smoking,^16^ who had a previous or current experience of shisha smoking, and were willing to participate in the study. A phenomenological approach was employed to explore lived experiences of shisha smokers.

### Selection of Participants

In the linked school survey^16^; 54 students reported previous or current history of shisha smoking and consented to a follow-up interview. These students were contacted by telephone and 10 students agreed to participate of which six participated in the actual interviews, three from Addis Ababa and three from Adama. Additional students were recruited using a snowball sampling technique by ensuring that we had the necessary range and diversity of participants that qualitative research seeks to achieve. This included a mix of students by age, sex, school grade, type of school, religion, and duration of shisha use experience. The combination of these recruitment methods resulted in a final purposive sample of 25 secondary school students.

### Data Collection Procedures

Interviews were conducted by the first author (SH) and two academic staff from Addis Ababa University, who have substantive experience in qualitative research. A one-day training session was provided to the team with a focus on the topic guide along with a refresher session on conducting qualitative interviews. Based on the study participants’ preferences interviews were conducted in the open air and quiet spaces of different hotels.

Data collection was undertaken from October 12-22, 2020. A topic guide was used to facilitate the in-depth interview which was developed by reviewing literature and translated to the local language (Amharic). Interviews varied between 40-90 minutes and a digital audio recorder was used to record the discussion. All interviews were conducted in Amharic, transcribed and anonymized, before being translated into English.

### Data Analyses

An inductive thematic approach, which facilitated reporting of participant experience, meaning, and reality^17^ was used. The research team read and re-read each transcript to familiarize themselves with the content of the interviews. A coding frame was prepared by SH and frequent discussion and refinements were made by the research team members FD and MK. Each transcript was coded using Atlas.ti version 8 software^18^ by two staff members from Addis Ababa University. Regular discussion between the coders and lead researcher (SH) took place to discuss any differences and to reach a consensus. The analytical approach was iterative, with interview transcripts analyzed at the time of coding and re-analyzed after a draft result section was written to search different themes in regards to the students’ lived experience of shisha smoking.

### Ethics Consideration

As this research is a continuation of the previous school survey, we sought consent from each schoolmaster for students less than the age of 18 years. All interviews were performed per the relevant guidelines and regulations set out in the Declaration of Helsinki.

## Results

A total of 25 students participated in both cities (13 from Adama and 12 from Addis Ababa). Seventeen students were men and 8 were women. The age of the students ranged from 15-22 years. All participants were students, except for one who dropped out in grade 10. Further respondent detail is presented in Table 1.

Results from our thematic analysis are presented under the following six themes; initiating shisha smoking, continued shisha smoking, source of funds to buy shisha, negative side effects of shisha smoking, concealment, and concerns about shisha use.

### Initiating Shisha Smoking

Students discussed two key factors that influenced their decision to start shisha smoking; peer influence and seeing it as a means to release stress.

#### Peer Influence

Peer influence was a dominant factor and had a key role in shisha initiation. For example, students described hearing what their friends said about shisha use and accompanied them to shisha houses to try shisha for themselves. The comment was also made that students did not want to disappoint their friends or be different from their peer group.

A regular shisha user said:

“I started smoking suddenly. I went to my friend’s house; (....) when I get there, they were smoking shisha and I wanted to try it. And when they asked me to do it (....) I wanted to say no but at the same time, it is something my friends were doing. They said that nothing would happen to you and has no problem, and passed to me the shisha so I had tried it like everybody”.

[An 18-year old male student, Addis Ababa]

#### Escapism

Another facilitating factor for shisha was an escape from stress. Students reported that they started using shisha to forget their life frustrations; which included negative childhood experiences and bad moods. Moreover, having a dispute with a close family member or a friend was a trigger for initiating shisha smoking. Previously, they heard about the nice feeling created by shisha use and when there was an incident that affected their mood; they would smoke shisha.

“(....) my friend used to ask me to smoke, but I used to tell her no. (......) start smoking because I was mad, that is how I started smoking”.

[A 19-year old female student, Addis Ababa]

### Continued Shisha Smoking

Once students were exposed to shisha smoking there were several reasons for continued use. These included: khat chewing; peer influence; enjoying the shisha itself and the environment; having a lot of leisure time; and having access to shisha.

#### Khat Chewing

Khat chewing was mentioned by the students as a reason for continuing shisha smoking.

The data showed that shisha smoking was strongly linked with khat chewing practice. It was said that shisha smoking complements the effect of khat and it was very common to smoke shisha after or in parallel with chewing khat. It was common for interviewees to state that they would not like shisha if they did not chew khat.

A regular shisha user for the past two years noted that:

“Just like cigarettes, you want to smoke shisha when you chew khat.... You do not normally think about smoking shisha unless you are chewing khat”.

[A 19-year old male student, Addis Ababa]

A regular shisha user added that:

“When you chew khat your eyes become wide open, then if you smoke cigarettes, shisha or if you drink “Areke” [local alcohol drink] then it will break the effect.

[A 15-year old male student, Addis Ababa]

In contrast to this, a student stated:

“We sometimes smoke shisha only when we don’t want to chew khat. There is no association with khat”.

[A 17-year old male student, Addis Ababa]

#### Peer Influence

Similar to starting shisha use, peer influence was also found to be an important factor in continued use. Students smoked shisha in a group and would have a high tendency to invite and encourage their friends to smoke. Students reported that it was difficult to say no to the invitation from their friends. A previous shisha user explained that the reason he smoked shisha for two years was to maintain his place in his peer group,

“Not because I like the shisha but I didn’t want to be different from my friends. (…….) I didn’t want to be separated from my friends so I would go with them to smoke. I would just say that I like it so that they won’t be mad. Even though I know the risk I would say it’s nice for the sake of them”

[An 18-year old male, Adama]

#### Enjoying Shisha Smoking and the Environment

Students also stated that enjoying shisha smoking itself and the environment were reasons for smoking shisha. Shisha smoking made them relax and forget stressful situations at least for a little while. They liked the shisha smoke flavours available such as apple, mint, and chocolate. Students also liked the shisha smoking environment where they can have a chance to meet, talk and laugh with different people.

A regular shisha smoker for two years stated that:

“When you inhale the shisha, you will feel the flavour like incense and it feels nice when you feel that in your mouth so you just want to keep smoking. You don’t want to pass it to the next person”.

[A 15-year-old male student, Addis Ababa]

#### Leisure Time

Having free time was reported as a reason for smoking shisha by the students. Many students complained that they didn’t have access to sports games and other recreational activities, and community youth centres were perceived as unorganized and unattractive to young people. This resulted in boredom and a preference to go to shisha houses.

Students from government schools who regularly smoked shisha noted that:

“I smoke and chew only once a week during school time, but now we don’t have class so I can’t spend all day at home it is stressful”

[A 20-year-old female student, Addis Ababa]

#### Accessibility of Shisha

Accessibility for shisha equipment was mentioned as a reason for continued shisha use. Students could rent or buy the equipment for shisha smoking. Once they bought the shisha equipment in a group, they are only expected to buy the *Moassel* (which is named locally *Bureau)* regularly. This makes it less expensive to smoke shisha. They can buy some amount of *Moassel* with 25 Ethiopian Birr (ETB) which is equivalent to US$0.5. This can be smoked for one session which lasts 20 minutes with a group of four smokers.

A student from grade 10 who had dropped out of formal education and who was using shisha regularly for four years stated that:

“I don’t have any expense because she [a friend] has it [the shisha equipment] at her house. For sure, I would not pay to smoke daily but since I have it at her house for free, I smoke it daily. If you have to pay for it, then you have to get money to meet your need so I would not have become addicted for sure if I had to pay for it”.

[19-year old female drop-out student, Addis Ababa]

### Sources of Funds to Buy Shisha

The expense for shisha smoking depends on the type of the shisha house, the number of shisha smoking sessions, and the number of shisha smokers in a group. Students usually smoke shisha in a small house with a group of three to five people, spending around three to four hours together each day. Students may spend 150 ETB (US$3) on shisha per day and they would have additional expenses for khat, water, coffee, and other substances. It was highlighted that ‘in the addiction world’ people share their money with their friends. In a group, if one person has money, they would cover expenses for others. Students stated that they would ask their parents for money in the name of different daily expenses and used that money to buy shisha. Some students had small jobs and others would sell different valuables, stealing from home to cover their shisha expenses in a time when the money they would get from family was not enough.

A student from a government school stated that

“I would just pressure my mom to give me the money, then I will come to shisha’s house; whether I got 50 or 100 ETB (US$1 or 2) from her. If she doesn’t give me, I will just come to shisha house and ask someone to offer me khat and shisha”.

[A 20-year-old female student, Addis Ababa]

Students described several ways that they funded their shisha use. For example, they would steal money from their mother’s purse or get involved in a robbery to cover their expenses for substances. A previous shisha user for two years acknowledged that;

“Yes, I started stealing to survive. We used to be bold, so we were not scared of anybody, so we used to take money from strangers. We used to make around 10,000 ETB (US$200) in a day and we will spend it in a day. We will give some to our friends. We spend maybe 1,000 ETB (US$20) for food and the remaining we use for our addiction. We will go from one club to another all night”.

[An 18-year old male student, Adama]

### Negative Side Effects of Shisha Smoking

Students reported that they had experienced headaches, light headiness, coughing, vomiting, difficulty of breathing, and even fainting during shisha smoking. Usually, these symptoms happened when someone takes a deep breath for a few minutes for initiating the shisha smoke or when someone smokes shisha for the first time. A regular shisha user explained:

“(…….) you will feel lightheaded. (....) the difficulty of breathing happens if you don’t say enough. For instance, if you smoke alone and if there is no one with you to share then you will have difficulty of breathing”

[A 19-year old male student, Addis Ababa]

Those students who regularly chewed khat explained that they could not sleep if they did not smoke shisha. This is because khat chewing causes sleeping problems and makes them hyperactive. Students who chew khat uses different substances such as cigarette, shisha, and alcohol to break the effect of khat in a short time. Mostly, students chewed khat first and then smoke shisha after that or they may use it simultaneously. A regular shisha user for eight years stated that:

“I won’t be able to sleep if I didn’t smoke shisha. I chew khat daily and if I don’t smoke shisha, I won’t’ be able to sleep”.

[A 19-year old female student in Adama]

### Concealment of Shisha Use

Students reported that they did not tell their parents about their shisha use. They explained that they use different tactics to cover up their shisha smoking. Students spent school time in shisha houses and return home wearing their uniforms as if they were coming from their school. When there was no school, they gave different reasons to go out from their home to a shisha house.

A regular shisha user for four years stated that:

“We would go out while they[parents] were sleeping or we would say that we are going to church. Sometimes they suspect, but I would wear my dress and scarf and pretend like I am going to church”.

[A 19-year old female student, Addis Ababa]

### Concerns About Shisha Use

Expressing feelings of regret about using shisha was common among students. This was spoken in the context of regretting fights they had with their families, poor academic performance, stealing money from parents, and wasting a lot of money. However, there was also concern about their future life prospects especially female students were concerned about getting pregnant.

Female students explained that after exposure to second-hand smoke of hashish in the small and unventilated room; their judgement and memory could be impaired. For this reason, they would be vulnerable to sexual assaults. Lack of parental care and gaps in law enforcement were reported as reasons for engaging in shisha use, which for some students appeared to justify their lack of accountably for using substances instead of attending school.

A grade 9 government school student said that:

“I should be able to read English by now, but I can’t. I know how to read Amharic, but I don’t know how to read English, and I get jealous when I see people younger than me read English. (…..). And if the police took action while they caught me instead of letting me go for 200 ETB (US$4), I may not have repeated the action, but now I will make sure that I have 200 ETB (US$4) at hand to give them in case I get caught”

[An 18 years old male student, Adama]

A regular shisha user for four years stated that:

“(…..) I think about what if this kind of thing[pregnancy] happens to me without my knowledge. My mother has lots of kids and I imagine bringing another child to her and I feel bad. And sometimes you even hate your life when you think about it. You think if you could just die and if you didn’t exist. You use death as an escape and most girls committed suicide”

[A 19-year old female student, Adama]

## Discussion

This study was conducted to explore the lived experience of shisha smokers among high school students. Findings demonstrated that peer influence played an important role in the initiation and regular use of shisha; other studies conducted in Sudan and Ethiopia support this finding.^3,10^ Unlike cigarette smoking, shisha is mostly smoked in groups and provides opportunities to socialize. This helps to explain why peer-influence was found to be such an important factor. Unfortunately, in Ethiopia, there is no education given at schools about the prevention of dependence on shisha or indeed other substances. This means that young people generally acquire information about shisha smoking from their peers which means they may not be aware of the negative health effects. Furthermore, experiencing stress, which could be the result of an argument with family members or close friends was mentioned as a reason for shisha use. Smoking shisha was perceived to be a coping mechanism that would enable them to forget stressful situations and enjoy the moment. These findings are similar to those from the systematic review of 56 studies which showed the main motives for school and University students to use shisha were entertainment, pleasure, relaxation, and socialization.^19^

Students in this qualitative study were smoking shisha to enhance the effect of khat. Khat is a stimulant plant that is easily accessible in urban areas where it is sold in a small shop near schools and higher education institutions. In Ethiopia, there is no law prohibiting the sales and use of khat for under-age groups. It is very common to see adolescents chewing khat to stay awake and for recreational reasons. This plant is attributed as being a gateway to tobacco products used in studies conducted in the UK, Middle East, and African countries.^19,20^ In addition, a qualitative study conducted in the northern part of Ethiopia, reported that shisha smokers were khat chewers.^10^ After chewing khat, people smoke shisha, drink alcohol, or smoke cigarette to enhance the effect. It is further reported that to break the effect of depression after the effect of khat decreased, cigarette or alcohol are being used.^21^ After chewing khat, instead of smoking cigarettes, students may prefer to smoke shisha. This is due to shisha smoke having a nice flavour and little odour which makes it easy to hide from their families.

Students stated that having leisure time was a reason for smoking shisha. When they had free time, there were very limited affordable places to visit where they could socialize and had fun. Shisha houses or khat shops were left as the only places to spend time with friends. Supporting this, another study in Ethiopia conducted in 2020 found that there were over 3,000 youth centres where only half of them were functional.^22^ Moreover, their contribution in terms of promoting positive youth development is minimal and even some were centres where youths acquire negative health behaviours.^22^ Youth-friendly centres where students can develop knowledge, skills and spend time with their peers should be expanded, accessible and affordable.

In this study, accessibility was mentioned as one reason for smoking shisha. Based on strong tobacco control laws in Ethiopia [Proclamation 1112/2019] it is forbidden to smoke and sell any kind of flavoured tobacco.^15^ However, it is evident to see shisha houses are everywhere. Also, students rent houses and shisha smoking equipment which made it easy for them to smoke regularly. This makes the police and other stakeholders’ job difficult to control the shisha smoking practice. Awareness should be created for the community that renting out houses for shisha smoking is forbidden in the law and implementation of the tobacco control law should be strengthened.

Based on this study, shisha smokers experienced headaches, cough, light-headedness, shortness of breath and even fainting while smoking. This mostly happened when they were trying to inhale the first smoke of shisha after putting it in burning charcoal. Most students mentioned that taking a deep breath to take the first smoke of shisha is difficult and needs a lot of energy. The charcoal that is used for shisha smoking is small and less flammable compared to the one used for the Ethiopian coffee ceremony. The symptoms could result due to inhaling the carbon monoxide while taking the first breath of the shisha smoke. Normally the shops for shisha smoking where students smoke shisha are small, suffocated, and with no or minimal ventilation spaces. If students were smoking in rented houses or even in the shisha shops, they would close any door or windows; so that no one would notice the smoke. In the session of shisha smoking, carbon monoxide intoxication can result in syncope due to secondary to the formation of carboxyhaemoglobin in the blood, which compromises the transportation of sufficient oxygen to body parts including the brain.^23^

We found that students hide their shisha smoking practice from their parents and school teachers. Students used different techniques to hide their smoking practice and mostly they were successful. The reason for hiding could be fear of not getting approval from their families or teachers. Hiding their practice narrows the possibility to get guidance and support from adults. Therefore, shisha smokers were doing what makes them happy and feels right for them and their peers. After becoming dependent and time passed, students regretted their lack of engagement with learning and academic performance, their communication with family, and other parts of their lives. Moreover, female students were vulnerable to reproductive health problems. Interventions are needed that raise the awareness of female students about reproductive health risks related to shisha use. A systematic review showed that school, family and community-based interventions had a positive impact on the prevention of smoking /tobacco use among adolescents.^24^

### Strengths and Limitations of the Study

This study is the first qualitative study to explore the lived experience of shisha smokers among high school students in the two main urban settings (Addis Ababa and Adama) of Ethiopia. We used the snowball sampling technique that has limitations in terms of recruiting similar students to those we initially identified via a wider survey in schools. To minimize this, maximum variability criteria were used in the identification and recruitment of the students. There could be social desirability bias where boys would overestimate and females underestimate their exposure and experience of shisha smoking. To keep down this, we encouraged students to tell us their actual experiences with shisha smoking.

## Conclusions

Our study found that peer influence plays a major role in high school students’ shisha smoking uptake and ongoing use. Furthermore, khat chewing, accessibility, and having free time were mentioned as reasons for regular shisha use. It is encouraging that Ethiopia has a strong tobacco control law, but without controlling khat use, it is difficult to control the use of shisha. Students regretted their shisha use experience which could contribute to poor academic performance and conflict within families. Students reported some experience of health problems that may be related to the carbon monoxide intake while shisha smoking. Furthermore, female students reported reproductive health risks that may be related to their shisha use. Awareness-raising campaigns and programs about the negative health effects of shisha use for secondary school students should be designed and implemented in Ethiopia, alongside further efforts to fully implement tobacco control laws that prohibit the availability of shisha.

## Figures and Tables

**Table 1:** Respondent’s characteristics.

Characteristics	Addis Ababa	Adama	Total
**Gender**			
Male	8	9	17
Female	4	4	8
**Age group**			
15-16	2	0	2
17-19	9	12	21
20-22	1	1	2
**Grade**			
9	2	4	6
10	8	7	15
11	1	2	3
School dropout from grade 10	1	0	1
**School type**			
Government	10	9	19
Private	2	4	6
**Shisha smoking experience (Duration of use)**			
Previous user (< 1 months)	1	2	3
Previous user (3 years)	0	1	1
Regular user (<=1 year)	1	2	3
Regular user (2 years)	5	4	9
Regular user (3^+^ years)	5	4	9
**Total**	**12**	**13**	**25**
